# Influence of the Amount of Instability on the Leg Muscle Activity During a Loaded Free Barbell Half-Squat

**DOI:** 10.3390/ijerph17218046

**Published:** 2020-10-31

**Authors:** Bernat Buscà, Joan Aguilera-Castells, Jordi Arboix-Alió, Adrià Miró, Azahara Fort-Vanmeerhaeghe, Javier Peña

**Affiliations:** 1 Faculty of Psychology, Education Sciences and Sport Blanquerna, Ramon Llull University, 08022 Barcelona, Spain; joanac1@blanquerna.url.edu (J.A.-C.); jordiaa1@blanquerna.url.edu (J.A.-A.); adriama@blanquerna.url.edu (A.M.); azaharafv@blanquerna.url.edu (A.F.-V.); 2 School of Health Science Blanquerna, Ramon Llull University, 08025 Barcelona, Spain; 3 Sport and Physical Activity Studies Centre (CEEAF), University of Vic–Central University of Catalonia, 08500 Vic, Spain; javier.pena@uvic.cat; 4 Sport Performance Analysis Research Group (SPARG), University of Vic-Central University of Catalonia, 08500 Vic, Spain

**Keywords:** unstable, perturbation, electromyography, squatting

## Abstract

This study aimed to understand the acute responses on the muscular activity of primary movers during the execution of a half-squat under different unstable devices. Fourteen male and female high-standard track and field athletes were voluntarily recruited. A repeated measures design was used to establish the differences between muscle activity of the primary movers, the body centre of mass acceleration and the OMNI-Perceived Exertion Scale for Resistance Exercise (OMNI-Res) in a half-squat under four different stability conditions (floor, foam, BOSU-up and BOSU-down). A significant correlation was found between the highest performance limb muscle activity and body centre of mass acceleration for half-squat floor (r = 0.446, *p* = 0.003), foam (r = 0.322, *p* = 0.038), BOSU-up (r = 0.500, *p* = 0.001), and BOSU-down (r = 0.495, *p* = 0.001) exercises. For the exercise condition, the half-squat BOSU-up and BOSU-down significantly increased the muscle activity compared to half-squat floor (vastus medialis: *p* = 0.020, d = 0.56; vastus lateralis: *p* = 0.006, d = 0.75; biceps femoris: *p* = 0.000–0.006, d = 1.23–1.00) and half-squat foam (vastus medialis: *p* = 0.005–0.006, d = 0.60–1.00; vastus lateralis: *p* = 0.014, d = 0.67; biceps femoris: *p* = 0.002, d = 1.00) activities. This study contributes to improving the understanding of instability training, providing data about the acute muscular responses that an athlete experiences under varied stability conditions. The perturbation offered by the two BOSU conditions was revealed as the most demanding for the sample of athletes, followed by foam and floor executions.

## 1. Introduction

Athletic performance is associated with specific neuromuscular adaptations improving the motor unit recruitment and the coordination of all the muscles involved in a given action. For such purposes, athletes perform different motor tasks searching for varied and effective training stimuli [1]. In this vein, a progression in load has been the ideal strategy for increasing muscular demands, but, in recent years, unstable environments have also been used with similar purposes [2,3,4]. Thus, different unstable devices have been used to enhance the effects of several exercises on muscle activation, force production, motor control, and consequently, athletic performance [1,5,6]. The design of these devices is intended to alter the relationship between the base of support, the body’s spatial position, and the athlete’s ability to maintain balance during the execution of a task. Therefore, the amount of instability depends on factors such as the nature of the task, characteristics of the subject (weight, height, muscle abilities and motor control) and the different features of the device (shape, material, friction, size and display) [1].

Performing conditioning exercises in an unstable environment, such as on a BOSU, Swiss balls, rubber discs, and freeman plates, or hanging loose objects on barbells, creates perturbations in whole-body stability. Thus, perturbation training represents a new challenge for somatosensory, vestibular, and visual systems [7]. Moreover, perturbed tasks increase the co-contractile activity, enhancing the role of antagonists to mitigate the uncertainty produced by the source of instability [8]. But how much instability does each device generate in the environment? Is this acute response the same for different athletic profiles? Which muscles are more demanded, and which are worked less when squatting? To address these questions, several studies have been conducted to assess the impact of instability on muscle activation during the execution of a squat [2,3,6,9,10]. As examined by Behm and Anderson [1], several authors have reported decrements of muscle activity of the primary squat movers under unstable conditions [3,6]. Specifically, McBride et al. [3] showed higher muscle activity of the vastus lateralis and biceps femoris under stable conditions (floor vs inflated disc) in three different loaded-squats in recreationally resistance-trained men, and Andersen et al. [6] found non-significant differences between stable and unstable squat conditions (foam) in the rectus femoris and both vastus muscles in males with a background in strength training. McBride et al. [2] found significantly higher muscle activity in the biceps femoris when squatting on two inflatable balance discs in recreationally resistance-trained males. However, no significant differences were found in both vastus muscles by Saeterbakken and Fimland (2013), when comparing the muscle activity of all the primary squat movers under different unstable conditions (Power Board, BOSU and Balance Cone). The authors established the instability properties of the devices used based on the number of unstable axes and the magnitude of contact with the floor. Unstable environments have been revealed in several studies to be a useful tool to elicit higher muscle activation in the core muscles when squatting [5,11,12].

As mentioned earlier, several groups of researchers have studied the acute responses of different unstable environments on muscle activation and force/power production in the past [3,10,13,14], but to the best of our knowledge, none of them have quantified the amount of instability created. According to the studies’ designs, it can be inferred that some devices can create higher instability than others, but no data are available describing how unstable every condition is. Other studies have reported data using accelerometers in strength and conditioning settings. Thus, Vazquez–Guerrero et al. [15] compared the force output under different stability conditions of a flywheel squat providing mean values, and a correlation between thigh muscle activation and mean acceleration of body centre of mass has been found by Aguilera–Castells et al. [16] in a suspended lunge under unstable dual conditions. In other contexts, Thiel et al. [17] used different accelerometers to assess the quality of the movements in professional dancers, with lower acceleration peaks associated with higher performance in a series of demi-pliés. Moreover, Johnston et al. [18] used an inertial sensor to detect minor changes when performing the Y Balance test in healthy adults, calculating the postural adjustment from the XY axis and filtered data from a gyroscope. In the tested task, participants were required to explore their limits in stabilizing the whole body. Additionally, Barbado et al. [19] used the accelerometer of a smartphone to describe the intensity of core training through the quantification of the centre of pressure mean linear acceleration in different unstable environments. Thus, associating muscular activation with the amount of instability at each repetition of a set of exercises under different unstable conditions could explain the real effect of the different sources of instability on athletes. Nevertheless, the methods used in the cited studies present insufficient or questionable validity in some movements because mean acceleration was considered, instead of the sum of the integrated (x- and y-axis) acceleration peaks.

Other investigations have quantified or altered the balance with sufficient validity and reliability using different methods such as force platforms [20], stabilometers [21] and pressure mats [22] in the context of ankle and knee rehabilitation processes, fall prevention and postural balance in different populations. However, all these methods have several limitations when assessing the amount of instability in dynamic strength and conditioning tasks. One of the main limitations when using force platforms, stabilometers or pressure mats together with a BOSU, foams, or other devices providing ground instability, arises from the fact that the base of support and the ground reaction forces significantly change with respect to the execution on the floor. Indeed, the devices’ characteristics change these parameters and, consequently, the validity of the amount of instability measured. Thus, measuring the dynamics of the body centre of mass far from the floor could address this issue. Understanding the amount of instability constitutes an essential factor in better explaining how challenging a task constraint is for the different sport profile [1]. In this regard, while some unstable environments are challenging for less-trained individuals, others are able to stabilize their posture even in the most unstable conditions. Therefore, muscle activity should reflect these differences.

Therefore, the first objective of the present study was to analyse the amount of instability in different half-squat conditions (floor, foam, BOSU-up and BOSU-down) experienced by high-standard athletes using an accelerometer, determining a protocol for its quantification. The second objective was to compare the muscle activity of the biceps femoris, vastus medialis, and vastus lateralis, and the global activity (sum of all the analysed muscles) of the highest performance limb during the execution of the half-squat and to assess the rating of perceived exertion (OMNI-Res) under the four aforementioned conditions. Thirdly, the relationship between the body centre of mass acceleration (BCMA) and the global muscle activity (sum of all the analysed muscles) was established. We hypothesized that the BOSU-down condition elicits higher BCMA than BOSU-up, foam and floor conditions, respectively, and follows this order of potential instability. In contrast, we expected lower muscle activity as the condition became more unstable. We also hypothesized a significant relationship between BCMA and global muscle activity, considering the different tested conditions.

## 2. Materials and Methods

### 2.1. Participants

Fourteen males (*n* = 5, mean age = 20.00 ± 1.41 years, range = 18–21 years; height = 1.73 ± 0.05 m, body mass = 64.00 ± 4.64 kg, body mass index = 21.48 ± 1.19 kg·m^−2^) and females (*n* = 9, mean age = 20.44 ± 1.67 years, range = 18–23 years; height = 1.67 ± 0.03 m, body mass = 56.72 ± 4.89 kg, body mass index = 20.29 ± 1.43 kg·m^−2^), all high-standard track and field athletes (i.e., 11 sprinters and 3 middle- distance runners), volunteered to participate in the study and were intentionally recruited. As inclusion criteria, all the participants were enrolled in a sport talent program, and all of them national finalists, training for at least 10 h per week (i.e., speed, endurance, and technical skill training) while engaging in international competitions. Participants were regularly checked by the sport talent program medical team, and none of them were excluded from the sample because they did not present any injury or pain related to cardiovascular, musculoskeletal or neurological disorders, following the American College of Sports Medicine exercise testing procedures. Before the familiarisation session and test session of the study, participants were encouraged to avoid consuming stimulants (e.g., caffeine), drinks or food 3 to 4 h before the session and to avoid high-intensity physical activity for 24 h before the test.

Before participating, each athlete was fully informed about the experimental procedures and the risks and benefits of participating in the study, as well as receiving and signing a written consent form. The Ethics and Research Committee Board in the Blanquerna Faculty of Psychology and Educational and Sport Sciences at Ramon Llull University in Barcelona, Spain, approved the study (ref. no. 1819005D). All protocols implemented in the study complied with the requirements specified in the Declaration of Helsinki (revised in Fortaleza, Brazil, 2013).

### 2.2. Experimental Procedures

A repeated measures design was applied to establish the relationship between muscle activity and body centre of mass acceleration (BCMA). Electromyographic activity, BCMA and results on the OMNI-Perceived Exertion Scale for Resistance Exercise (OMNI-Res) were compared during the resistance half-squat under different conditions of stability. The study was conducted in two sessions—familiarisation and test sessions—performed a week apart: both from 11 a.m. to 2 p.m. Firstly, the familiarisation session was conducted to acquaint the participants with the exercise technique and determine the highest performance limb, and the load lifted in a single maximum repetition (1 RM) in the half-squat. Secondly, the test session was used to assess muscle activity, BCMA and OMNI-Res results when performing the half-squat on four surface conditions: the floor, foam (Balance Pad; Airex, Sins, China), BOSU-up (BOSU, Ashland, OH, USA) with the dome side up, and BOSU-down with the dome side down.

The familiarisation session was held to collect the participants’ age, weight, height, leg length, the width of the distance between the anterior superior iliac spine, and other descriptive variables (e.g., hours of training). Next, a general 10 min warm-up was performed (i.e., squatting exercise with bodyweight, dynamic stretches and joint mobility of the lower limb involved in the half-squat exercise) and a specific 10 min warm-up consisting of one set of 20 repetitions of the half-squat with the additional load of the squat bar (10 kg) and two sets of 10 repetitions of half-squats with a loaded bar (60–70% 1 RM). Before the values of 1 RM were recorded, participants performed a unilateral half-squat against an invincible resistance to determine their maximum voluntary isometric contraction in the concentric phase, measured with two force sensors anchored to the ground. To individualise the exercise but to allow all participants to apply force with knee flexion of 90°, two non-elastic straps were anchored between the force sensor and the bar following Saeterbakken and Fimland’s protocol [10], used to establish the leg to be analysed under the different conditions of the exercise. The selected criterion was the highest-performing limb [23], defined as the side with the highest value in a specific task—in the study, the half-squat exercise. During the 1 RM test, the speed of the bar was controlled with a linear positional transducer (Chronojump-Boscosystem; Barcelona, Spain). During the warm-up, the velocity for the unloaded half-squat was determined to be >1.28 m·s^−1^ (<40% 1 RM) and for the loaded half-squat from 1.00 m·s^−1^ to 0.84 m·s^−1^ (60–70% 1 RM). To determine the value of 1 RM, participants performed a set of 10 repetitions of the half-squat on the floor condition, and according to the average speed and predictive equation *Load* (% 1 *RM*) = −5.961 *MPV2* − 50.71 *MPV* + 117.0, in which *MPV* refers to “mean propulsive velocity” [24], the value of each participant’s load was individualised according to the relative value of 80% of 1 RM. That load value (i.e., 80% 1 RM) obtained for the half-squat on the floor condition was used for all exercise conditions.

**Figure 1:**
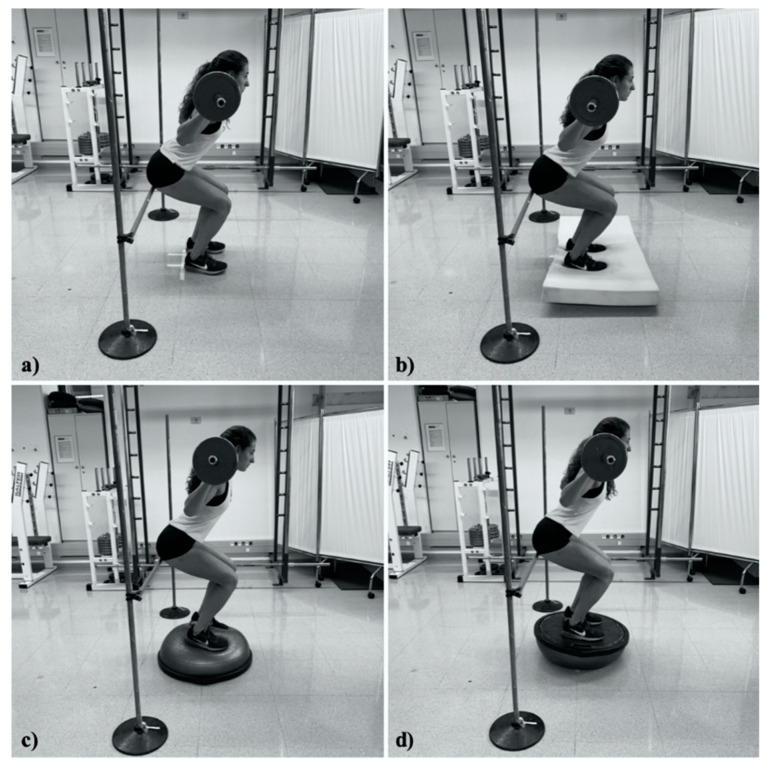
Exercise conditions: (**a**) half-squat floor, (**b**) half-squat foam, (**c**) half-squat BOSU-up, and (**d**) half-squat BOSU-down.

The test session began with placing electromyographic electrodes (BIOPAC EL504 disposable Ag–AgCl) with an inter-electrode distance of 2 cm on the vastus medialis, the vastus lateralis and the biceps femoris of the highest-performing leg according to the recommendations of the SENIAM project. Before placement, the leg was shaved, exfoliated, and cleaned with alcohol to reduce the impedance of dead tissue surfaces and oils. Afterwards, a tri-axial accelerometer was placed on the waist for measuring the BCMA. Then, participants performed a standardised warm-up involving dynamic stretching, joint mobility and squatting in a set of 10 repetitions at 40% 1 RM. Next, participants began performing the half-squat protocol on the four surface conditions (i.e., floor, foam, BOSU-up and BOSU-down) in a random order (Figure 1). In each condition, they completed a set of five repetitions with a relative load of 80% of 1 RM at 60 beats per minute at an eccentric-to-concentric phase ratio of 1:1. A linear positional transducer used to control the range of movement in all repetitions of the different surface conditions was attached to the participant’s hip. Between performing the half-squat exercise in each condition, participants received a 2 min rest period to prevent fatigue. Trials not performed with the proper technique were discarded and repeated.

The half-squat depth was normalised to 75% of the participant’s leg length, with the feet placed apart slightly wider than shoulder width and with toes pointed forward. The bar was placed across the shoulders on the trapezius slightly above the posterior aspect of the deltoids. Customised stoppers, similar to hurdles, were used to fix the lower position of the half-squat (Figure 1). Participants were instructed about the squat depth and when to commence the countermovement. Feedback regarding when to begin the half-squat and how to stand on the surface (i.e., upright, both feet planted and hands on the bar in a prone position) was provided. Participants’ shoulders were placed at 90° of abduction with a slight external rotation, while the lower back maintained a neutral position. Participants lowered their body (i.e., eccentric phase) until their gluteus touched the customised stoppers and subsequently returned to the starting position with a full knee extension of the legs (i.e., concentric phase).

### 2.3. Surface Electromyography Signals

The data acquisition system BIOPAC MP150 was used to record all the surface electromyographical values at a sampling rate of 1.0 kHz, and these data were analysed using the AcqKnowledge 4.2 software (BIOPAC System, INC., Goleta, CA, USA). The electromyographical surface signals were bandpass filtered at 10–500 Hz utilizing a fourth order Butterworth filter. For each exercise, the root mean square surface electromyography signals were recorded.

The surface electromyography signals of all the exercise conditions were analysed by taking the average of the three middle repetitions, excluding the first and fifth repetition from the data analysis. The surface electromyography signal amplitude in the domain was quantified using the root mean square, and these values were selected for every trial. The global mean of all muscles (i.e., vastus medialis, vastus lateralis, and biceps femoris) was calculated (arithmetic mean), and the global activity (sum of the three analysed muscles) was also calculated.

### 2.4. Body Centre of Mass Acceleration

All BCMA values were measured by a tri-axial accelerometer TSD109 F (BIOPAC System, INC., Goleta, CA) with a sample rate of 2.0 kHz, a sensitivity of 40 mV/g, and a range of ± 50 g. Data were collected using BIOPAC MP150 and the AcqKnowledge 4.2 software. The tri-axial accelerometer was calibrated according to the manufacturer’s recommendations.

**Figure 2:**
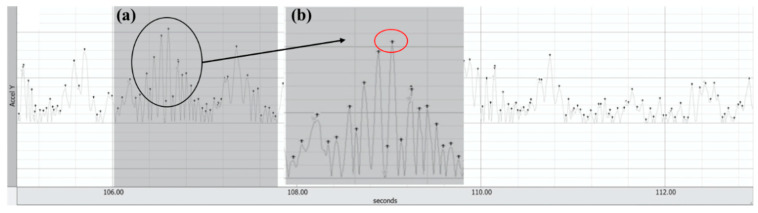
Body centre of mass acceleration signal (Y–axis). The signal shows all the changes in the body centre of mass acceleration (BCMA) during one repetition (entire phase) of the half-squat performed on the floor (**a**) = The shaded area shows the total number of amplitudes in the entire phase; (**b**) the magnified zone details each of the maximum BCMA values (red circle). These values were summed to determine the value of BCMA in the entire phase.

Before analysing data from the BCMA, a bandpass filter fixed at 0.5 Hz (low), and 20 Hz (high) was applied, and then this signal was integrated with a root mean square. The BCMA values were analysed using the complete repetition on the anterior–posterior and proximal–lateral axes. The first and fifth repetitions were excluded from data analysis. The sum of amplitudes in the entire phase was analysed (Figure 2). This data analysis was based on the sum of all the maximum BCMA values reached in the entire phase. The global mean of the BCMA for each axis under all the exercise conditions was calculated. Next, the vector of acceleration was calculated as the quadratic combination of the global mean values of the anterior–posterior and proximal–lateral axes. After that, the global mean of this vector (arithmetic mean) was also calculated and analysed. This calculation method reflects the magnitude of the micro destabilizations necessary to maintain a balanced posture while squatting. A mean of all the acceleration data does not reflect this phenomenon, while the sum of peak values does.

### 2.5. OMNI-Perceived Exertion Scale for Resistance Exercise

The OMNI-Res was used to rate the perceived exertion of the participants for each half-squat condition. Participants were asked to rate their perceived exertion for the overall body on completion of each exercise. During the familiarization session, participants were instructed to assign a rating of 1 to any perception of exertion that was less than that experienced during the unweighted repetition and a rating of 10 to any perception of exertion that was greater than that experienced during a 1 RM lift. The assessment of the OMNI-Res during the testing session was conducted following the Robertson et al. [25] instructions. Moreover, all the participants were instructed to establish a visual–cognitive link depicted visually by an athlete lifting weights at the top and bottom of the OMNI-Res scale. After collecting OMNI-Res values, the global mean of OMNI-Res (arithmetic mean) was calculated and analysed.

### 2.6. Statistical Analysis

The number of participants chosen was based on effect size 0.40 SD with an α level of 0.05 and power at 0.95, using G Power Software (University of Dusseldorf, Dusseldorf, Germany). The Shapiro–Wilk test was used to confirm that data were normally distributed to approve the use of the parametric techniques. The results were analysed by a statistical description of each of the dependent variables to obtain the mean values and standard error of the mean (SE) (mean ± SE). The intra-rater reliability of all quantitative dependent variables (muscle activity and BCMA) was assessed using an intraclass correlation coefficient (ICC), and their 95% confidence intervals were based on a mean rating (K = 3), absolute agreement, and a two-way mixed-effects model. Pearson’s correlation (r) was used to determine the relationship between muscle activity (global activity) and BCMA of each repetition and exercise condition. Moreover, a linear mixed model analysis was used for global activity and included the exercise condition (half-squat on floor, foam, BOSU-up and -down) and BCMA as fixed effects, and participants were considered as random effects. The effect of every exercise condition on muscle activity (vastus medialis, vastus lateralis, biceps femoris, and global activity) was analysed using a linear mixed model, which was fitted to analyse whether the changes for muscle activity were influenced by exercise condition. The activation of vastus medialis, vastus lateralis, biceps femoris, and global activity were considered to be the dependent variables, the exercise condition (floor, foam, BOSU-up, and BOSU-down) was considered as a fixed effect, and participants were considered as random effects. Furthermore, another linear mixed model was used to examine whether the exercise condition modified the BCMA; the BCMA was considered as the dependent variable, the exercise condition (floor, foam, BOSU-up, and BOSU-down) was considered as a fixed effect, and participants were considered as random effects. For the previous models, the significance of the fixed effects associated with the outcome variable included in the model was assessed using the Wald test, with statistical significance set at *p* < 0.05. After the models were validated, the residuals of the final models were explored for normality, homogeneity, and independence assumptions. The normality assumption of the residuals was checked using a normal Q–Q plot of residuals. The OMNI-Res data did not meet the inferential parametric assumptions. A non-parametric Friedman test was used to examine the effect of exercise on the OMNI-Res. Post hoc Wilcoxon test analysis with Bonferroni correction was used in case of significant main effects. For pairwise comparison, the Cohen’s *d* effect size was calculated [26], and the magnitude of the effect size was interpreted as <0.2 = trivial, 0.2–0.6 = small, 0.6–1.2 = moderate, 1.2–2.0 = large, and >2.0 = very large [27]. The ICC was interpreted using the recommendations of Koo and Li [28], i.e., poor (<0.5), moderate (0.5–0.75), good (0.75–0.90), and excellent (>0.90) reliability. Likewise, the magnitude of the Pearson’s correlation values was interpreted as < 0.1 = trivial, 0.1–0.3 = small, 0.3–0.5 = moderate, 0.5–0.7 = large, 0.7–0.9 = very large, and 0.9–1 = nearly perfect. Statistical data were analysed using SPSS (Version 26 for Mac; SPSS Inc., Chicago, IL, USA) with a significance value of *p* < 0.05.

## 3. Results

**Figure 3:**
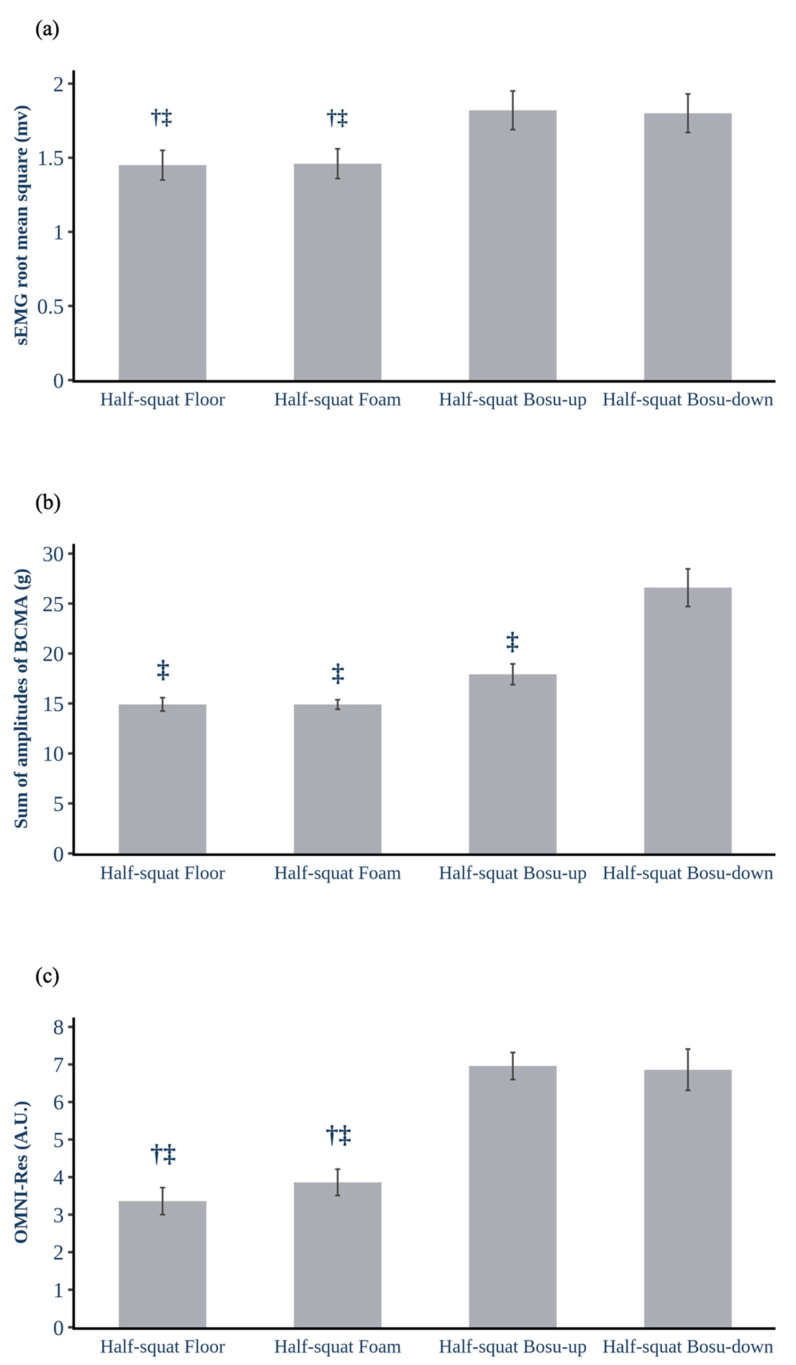
Comparison of the collected data under half-squat conditions: (**a**) global activity ^§^, (**b**) body centre of mass acceleration, and (**c**) OMNI-Perceived Exertion Scale for Resistance Exercise (OMNI-Res). Each bar represents the mean, and the error bar represents the standard error of the mean (SE). ^§^ = Sum of the activity of the vastus medialis, lateralis and biceps femoris; sEMG = surface electromyography; mV = microvolts; BCMA = body centre of mass acceleration; A.U. = Arbitrary units; † Significantly different from half-squat BOSU-up; ‡ Significantly different from half-squat BOSU-down.

The ICC demonstrated good to excellent reliability under all exercise conditions for all the analysed muscles and BCMA values (Table 1). The Pearson correlation between the highest performance limb activity and BCMA was significant for half-squat floor (r = 0.446, *p* = 0.003), foam (r = 0.322, *p* = 0.038), BOSU-up (r = 0.500, *p* = 0.001), and BOSU-down (r = 0.495, *p* = 0.001) exercises, all of them with a moderate effect (r = 0.3 to 0.5). Additionally, the linear mixed model showed a significant fixed effect for exercise condition [F (3,42) = 6.706, *p* = 0.001] and BCMA [F (1,46) = 19.209, *p* = 0.000] on global activity (Table 2). The effect of exercise condition on muscle activity showed a significant fixed effect for exercise condition on vastus medialis [F (3,42) = 6.350, *p* = 0.001], vastus lateralis [F (3,42) = 6.039, *p* = 0.002], biceps femoris [F (3,42) = 10.051, *p* = 0.000] and global activity [F (3,42) = 10.028, *p* = 0.000], and the results from linear mixed model are shown in Table 3. Post-hoc analysis showed a significantly greater vastus medialis activity for half-squat BOSU-up than half-squat floor (*p* = 0.020, d = 0.56) and foam (*p* = 0.005, d = 0.60) exercises, and vastus medialis recruitment was also significantly greater for half-squat BOSU-down than half-squat foam (*p* = 0.037, d = 0.53) lifts. A significantly greater activity for vastus lateralis was achieved under the half-squat BOSU-down condition compared to half-squat floor (*p* = 0.006, d = 0.75) and foam (*p* = 0.014, d = 0.67) repetitions. For the biceps femoris, activity was significantly greater for the half-squat BOSU-up and half-squat BOSU-down than for the half-squat floor activities (*p* = 0.000, d = 1.23; *p* = 0.006, d = 1.00, respectively). Moreover, the biceps femoris activity was significantly greater for the half-squat BOSU-up than half-squat foam exercises (*p* = 0.002, d = 1.00) (Table 4). The global activity was significantly greater for half-squat BOSU-up than floor (*p* = 0.001, d = 0.85) and foam (*p* = 0.002, d = 0.83) repetitions, also this activity significantly increased for half-squat BOSU-down in comparison with half-squat floor (*p* = 0.003, d = 0.84) and foam (*p* = 0.004, d = 0.79) movements (Figure 3a).

Table 5 shows the results of the linear mixed model between exercise condition and BCMA; a significant fixed effect for exercise condition [F (3,42) = 30.873 *p* = 0.000] was found on BCMA. The BCMA was significantly higher for the half-squat BOSU-down than half-squat floor (*p* = 0.000; d = 2.22), foam (*p* = 0.000; d = 2.28) and BOSU-up (*p* = 0.000; d = 1.53) (Figure 3b). For OMNI-Res, the exercise condition showed a significant main effect [X^2^ (3) = 35.667 *p* = 0.000], and the OMNI-Res was significantly higher for half-squat BOSU-up and BOSU-down than half-squat floor (*p* = 0.006, d = 2.66; *p* = 0.008, d = 2.01, respectively) and foam (*p* = 0.005, d = 2.32; *p* = 0.009, d = 1.74, respectively) (Figure 3c). The raw data of this study is available as supplementary material.

## 4. Discussion

The first objective of the present study was to quantify the amount of instability in a half-squat using an accelerometer. The use of mean acceleration values might not be the best way to describe the amount of instability [15,19], and mean, or peak root mean square acceleration values do not reflect the ability to maintain the posture, because the moments when the participants are balanced are taken into consideration for the calculations [17]. Therefore, the sum of the peaks (Figure 2), considering the quadratic combination of the acceleration in anteroposterior and mid-lateral axes [29], seems to provide an accurate approach for quantifying the amount of instability (BCMA) in different unstable resistance training environments [30]. As expected, the results of the present study showed an increased BCMA from foam to BOSU-down conditions, and significant differences between all conditions and BOSU-down. The data also reflected differences between the two most stable conditions (floor and foam) and BOSU-up. This finding contributes to understanding the magnitudes of stability that a trained athlete experiences during the half-squat exercise on different unstable surfaces. The perturbation offered by the BOSU-down was the greatest, followed by the BOSU-up and the foam, and agreed with the Seaterbakken and Fimland [10] criteria to establish the magnitude of instability (unstable dimensions and magnitude of contact with the floor). Therefore, the BCMA does reflect how challenging it is for athletes to maintain their posture under the tested conditions, confirming the first hypothesis.

The second objective was to compare the global muscle activity, the rating of perceived exertion, and the BCMA during the execution of the half-squat under the four conditions. The analysis of variance showed a significant main effect for the three variables. The behaviour of muscle activity and OMNI-Res was similar, and significant differences were found in the BOSU compared to the floor and foam conditions. According to Andersen et al. [6], this study did not find significant differences in global muscle activation between stable and foam conditions. Moreover, although the authors reported differences in power and force outputs, Drinkwater et al. [14] found no significant differences between the foam and stable conditions in a loaded squat. However, when the load increased (100% of 1 RM), the foam condition became more ‘stable’, and the force output was higher in respect to other more unstable conditions (i.e., BOSU). In line with the studies mentioned earlier, the present results showed that the inclusion of foam pads during a squat might not be worthwhile for high-standard athletes, at least for increasing the activity of the knee extensor muscles. Furthermore, the use of high loads seemed to play a stabilizing role under unstable conditions, allowing higher muscle activation and, therefore, higher force production [10,14]. In the present study, participants squatted with extra loads corresponding to their body mass, which could have helped in stabilizing the posture and, consequently, perform higher muscle activity. Therefore, the second hypothesis was not confirmed in the athletes studied in the present investigation.

In recent years, the use of BOSU as a high-demand, unstable environment in strength and conditioning exercises has undoubtedly become widespread. BCMA described in the present study clearly shows the magnitude of the differences between BOSU and stable or foam conditions. In terms of muscle activity, the effects of performing squats on BOSU apparatus are unclear. Although McBride et al. [2] found lower muscle activity in the vastus lateralis and vastus medialis with similar unstable devices (Dyna Disc) in students, Saeterbakken and Fimland [10] found no significant differences in the same muscles comparing the stable condition with a Power Ball, BOSU, or Balance Cone in the same muscles in experienced resistance training participants. In contrast, the present study found higher muscle activity of the vastus lateralis and vastus medialis on the BOSU when compared to the more stable conditions (floor and foam). In the same vein, other studies [3,4,6], showed no significant lower-muscle activity in the biceps femoris under unstable conditions. Nevertheless, in line with Saeterbakken and Fimland [10], the present study found higher activity of this muscle in BOSU conditions. The experience of the athletes in the present study and their ability to maintain balance, even in the most perturbed conditions, might explain these differences. The contemporary trend of introducing unstable environments in training programs for experienced athletes might change the inhibiting effect of instability on the primary squat movers, and become a challenge for intramuscular coordination in highly trained and coordinated populations. Thus, using unstable resistance training exercises would force accommodation to an unstable environment, diminishing the loss of force and the extent of co-contractions [31]. Indeed, the present study was carried out with athletes who were able to perform squats in different conditions with good and excellent reliability scores (Table 1). Firstly, results confirmed the ability of the athletes to maintain the balanced posture in all conditions, including the most perturbed ones on the BOSU. Concretely, the BOSU-down condition presented the highest BCMA, but the athletes showed good and excellent reliability in both axes. These data reflect the excellent motor control of the athletes maintaining the posture in all conditions.

Regarding the differences in muscle activation between the two BOSU conditions, the present study found that the vastus medialis showed significantly higher activation in both BOSU conditions. It could be speculated that the tendency to avoid the dynamic knee valgus explains this finding. Indeed, although the BOSU-down condition created higher global instability, it offered a flat and rigid surface that compelled the participants to act differently in avoiding the knee valgus position. Although this study did not test this muscle, the role of the gluteus medius in stabilizing the posture can probably explain the lower activation of the vastus medialis in the BOSU-down [13,32,33] actions. Furthermore, the role of the biceps femoris co-contraction in the most unstable conditions seemed to be clear in a half-squat. In contrast to other studies [3,6,10], this study found significant increases in biceps femoris activation in the two BOSU conditions in comparison to the more stable conditions (floor and foam). The reason could be that BOSU creates higher anteroposterior instability. Only Saeterbakken and Fimland [10] used a BOSU, but the standard of their participants might explain the different findings.

The use of ratings of perceived exertion in resistance training exercises (OMNI-Res) is increasing. Its validity in terms of metabolic resistance training [25] and velocity-based training [34] has been pointed out. However, the relationship between perceived exertion and unstable environments is not clear [35,36]. The cited research investigated the effects of instability on bench press rating of perceived exertion in a trained population, but no research has studied the relationship between the amount of instability and muscle activity. In the present study, the OMNI-Res reflected similar increases to those in muscle activity throughout all the conditions. BCMA was slightly different concerning perceived exertion. The effect of performing a half-squat on a BOSU (up or down) caused almost the same perception of exertion, but the BOSU-down condition showed significantly higher BCMA than the BOSU-up condition. Therefore, beyond the instability role of the BOSU position demonstrated by the sum of BCMA values, muscle activity, and perceived exertion remained unchanged in both more unstable conditions (Figure 3).

There are several limitations to the present study. The particular characteristics of the sample, demonstrating high neuromuscular performance, prevents extrapolation of the results to the general population. The sample size, although the statistical power is acceptable, was also limited, as too was the number of analysed muscles. Further research should analyse the role of the stabilizers (e.g., gluteus maximus and medius, rectus abdominis, adductors, erector spinae) in the different conditions. Additionally, the present study was conducted using dynamic half-squats at 60 beats per minute. This controlled pace allowed an efficient and balanced execution, but the present results cannot be generalized to other rhythms and, of course, other motor skills. Further investigations should study this effect at different velocities and with explosive actions. Thus, the feedback provided in velocity-based resistance training might be complemented with BCMA data, monitoring how stable each repetition is. To summarize, the main strengths of the present proposal showed that the amount of instability can be quantified simply and suitably, especially on unstable surfaces, because nothing interferes with the relationship between the floor and the unstable device. In contrast, only the BCMA has been taken into account, but no acceleration measurements were obtained from other body parts such as the knee or the ankle. The data processing still requires the development of a proper algorithm for obtaining the BCMA in real time, while executing the movements.

## 5. Conclusions

The present study showed a higher muscle activity of the vastus lateralis, vastus medialis, and biceps femoris in BOSU conditions. This study contributes to understanding the magnitudes of stability that an athlete experiences during the squat exercise on different unstable surfaces. Moreover, OMNI-Res does not reflect the different level of perturbation (BCMA) found for the two BOSU positions, but this scale approximates the muscle activity of the primary movers in the studied half-squat conditions. Muscle activity in the primary half-squat movers increased under unstable conditions in elite athletes. These findings are in contrast to previous studies demonstrating insignificant differences between stable and unstable settings in this exercise. Experienced athletes and trained individuals showed different responses under unstable environments from those observed in other populations. Thus, the use of devices generating instability should be considered when the main objective is to increase the activity of the primary movers in this exercise and, potentially, in other exercises with similar muscular requirements. Therefore, the use of unstable conditions in strength and conditioning programs may increase variability, a crucial element to maintain chronic adaptations in long-term resistance training programs. Challenging experienced athletes by making their environment less stable seems to be a proper strategy to increase the acute responses and effects of lower-body resistance training. Nevertheless, the devices aimed at creating the mentioned challenging environments should be chosen accordingly to the ability of the individuals to control the movement while maintaining a balanced posture. Only by following this premise can the primary muscles be further activated to achieve better training effects. Thus, determining a BCMA limit could clarify how balanced the execution of a strength and conditioning exercise is, and the potential acute responses of the neuromuscular system. Moreover, monitoring the BCMA could be interesting in providing real-time feedback and quantifying the amount of instability in professional strength and conditioning contexts. The conclusions mentioned above, although in a very specific population, open up new possibilities in the fields of injury prevention and rehabilitation. As unilateral training revealed an essential element to be balancing the hamstring/quadriceps ratio, understanding which exercises generate more muscle activation when instability is a factor, and under what conditions they do so, allows a better prescription.

## Figures and Tables

**Table 1:** Reliability values for each muscle analysed and body centre of mass acceleration under half-squat conditions.

	Exercise Condition	ICCs (Level of Reliability)	95% CI		SEM
			Lower	Upper	
Vastus medialis	Half-squat Floor	0.827 (Good)	0.57	0.94	0.11
	Half-squat Foam	0.934 (Excellent)	0.84	0.97	0.06
	Half-squat BOSU-up	0.859 (Good)	0.65	0.95	0.11
	Half-squat BOSU-down	0.772 (Good)	0.45	0.92	0.09
Vastus lateralis	Half-squat Floor	0.939 (Excellent)	0.85	0.98	0.06
	Half-squat Foam	0.816 (Good)	0.56	0.94	0.09
	Half-squat BOSU-up	0.846 (Good)	0.63	0.95	0.11
	Half-squat BOSU-down	0.820 (Good)	0.57	0.94	0.12
Biceps femoris	Half-squat Floor	0.937 (Excellent)	0.85	0.98	0.02
	Half-squat Foam	0.952 (Excellent)	0.89	0.98	0.02
	Half-squat BOSU-up	0.946 (Excellent)	0.87	0.98	0.04
	Half-squat BOSU-down	0.886 (Good)	0.70	0.96	0.05
Y-axis acceleration	Half-squat Floor	0.960 (Excellent)	0.90	0.99	0.47
	Half-squat Foam	0.792 (Good)	0.49	0.93	0.74
	Half-squat BOSU-up	0.859 (Good)	0.66	0.95	0.90
	Half-squat BOSU-down	0.908 (Excellent)	0.77	0.97	1.37
X-axis acceleration	Half-squat Floor	0.953 (Excellent)	0.89	0.98	0.49
	Half-squat Foam	0.843 (Good)	0.62	0.95	0.64
	Half-squat BOSU-up	0.919 (Excellent)	0.81	0.97	1.15
	Half-squat BOSU-down	0.830 (Good)	0.58	0.94	2.94

95% CI = 95% confidence interval; ICCs = Interclass correlation coefficients; SEM = Standard error of measurement.

**Table 2:** Linear mixed model with exercise condition and BCMA as the fixed effects and global activity as the dependent variable.

	Parameter	ES	SE	95%CI		Test (df)	*p*
				Lower	Upper		
Global activity	Intercept	0.83	0.24	0.35	1.31	t (54) = 3.460	0.001
	Half-squat Floor	0.76	0.12	−0.17	0.32	t (45) = 0.620	0.539
	Half-squat Foam	0.09	0.12	−0.15	0.34	t (45) = 0.728	0.470
	Half-squat BOSU-up	0.34	0.10	0.12	0.55	t (44) = 3.229	0.002
	BCMA	0.03	0.01	0.02	0.05	t (46) = 4.383	0.000
	σ_u_	0.30					
	σ_є_	0.20					

ES = coefficient estimate; SE = standard error; 95% CI = 95% confidence intervals; df = degrees of freedom; t = t–value; *p* = *p*–value; BCMA = body centre of mass acceleration; σ_u_ = standard deviation of participant; σ_є_ = standard deviation of residual. We have used “half-squat BOSU-down” in the exercise condition variable as reference categories for this model.

**Table 3:** Linear mixed model with exercise condition as the fixed effects and muscle activity (vastus medialis, vastus lateralis, biceps femoris, and global activity) as the dependent variable.

	Parameter	ES	SE	95%CI		Test (df)	*p*
				Lower	Upper		
Vastus medialis	Intercept	0.73	0.06	0.60	0.85	t (20) = 12.116	0.000
	Half-squat Floor	−0.09	0.04	−0.17	−0.01	t (42) = −2.393	0.021
	Half-squat Foam	−0.11	0.04	−0.19	−0.03	t (42) = −2.886	0.006
	Half-squat BOSU-up	0.03	0.04	−0.05	0.11	t (42) = 0.721	0.475
	σ_u_	0.19					
	σ_є_	0.10					
Vastus lateralis	Intercept	0.74	0.05	0.63	0.84	t (25) = 14.605	0.000
	Half-squat Floor	−0.15	0.04	−0.24	−0.06	t (42) = −3.532	0.001
	Half-squat Foam	−0.14	0.04	−0.22	−0.05	t (42) = −3.236	0.002
	Half-squat BOSU-up	−0.03	0.04	−0.12	0.05	t (42) = −0.821	0.416
	σ_u_	0.15					
	σ_є_	0.11					
Biceps femoris	Intercept	0.33	0.02	0.27	0.38	t (31) = 11.875	0.000
	Half-squat Floor	−0.09	0.02	−0.15	−0.04	t (42) = −3.519	0.001
	Half-squat Foam	−0.07	0.02	−0.13	−0,02	t (42) = −2.763	0.008
	Half-squat BOSU-up	0.03	0.02	−0.02	0.08	t (42) = 1.199	0.237
	σ_u_	0.07					
	σ_є_	0.07					
Global activity	Intercept	1.79	0.11	1.56	2.02	t (24) = 16.115	0.000
	Half-squat Floor	−0.34	0.09	−0.53	−0.16	t (42) = −3.794	0.000
	Half-squat Foam	−0.33	0.09	−0.51	−0.14	t (42) = −3.645	0.001
	Half-squat BOSU-up	0.02	0.09	−0.15	0.21	t (42) = 0.297	0.768
	σ_u_	0.33					
	σ_є_	0.24					

ES = coefficient estimate; SE = standard error; 95% CI = 95% confidence intervals; df = degrees of freedom; t = t–value; *p* = *p*–value; σ_u_ = standard deviation of participant; σ_є_ = standard deviation of residual. We have used “half-squat BOSU-down” in the exercise condition variable as reference categories for this model.

**Table 4:** Root mean square surface electromyography values (mV) for each muscle analysed under half-squat conditions. Values are expressed as mean ± standard error of the mean (SE)**.**

	Half-Squat Floor	Half-Squat Foam	Half-Squat BOSU-Up	Half-Squat BOSU-Down
Vastus medialis	0.63 ± 0.06 ^†^	0.61 ± 0.06 ^†‡^	0.76 ± 0.07	0.73 ± 0.06
Vastus lateralis	0.59 ± 0.04 ^‡^	0.60 ± 0.05 ^‡^	0.70 ± 0.06	0.74 ± 0.07
Biceps femoris	0.23 ± 0.03 ^†^^‡^	0.25 ± 0.03 ^†^	0.36 ± 0.03	0.33 ± 0.03

mV = microvolts; ^†^ Significantly different from half-squat BOSU-up; ^‡^ Significantly different from half-squat BOSU-down.

**Table 5:** Linear mixed model with exercise condition as the fixed effects and BCMA as the dependent variable.

	Parameter	ES	SE	95%CI		Test (df)	*p*
				Lower	Upper		
BCMA	Intercept	26.59	1.10	24.37	28.81	t (50) = 24.043	0.000
	Half-squat floor	−11.69	1.41	−14.52	−8.85	t (42) = −8.307	0.000
	Half-squat foam	−11.69	1.41	−14.53	−8.85	t (42) = −8.309	0.000
	Half-squat BOSU-up	−8.67	1.41	−11.51	−5.83	t (42) = −6.166	0.000
	σ_u_	1.80					
	σ_є_	3.72					

ES = coefficient estimate; SE = standard error; 95% CI = 95% confidence intervals; df = degrees of freedom; t = t-value; *p* = *p*-value; σ_u_ = standard deviation of participant; σ_є_ = standard deviation of residual. We have used “half-squat BOSU-down” in the exercise condition variable as reference categories for this model.

## Supplementary Materials

The following materials are available online at https://www.mdpi.com/1660-4601/17/21/8046/s1, Spreadsheet S1: Raw data.

## References

[1] Behm D., Anderson K. (2006). The role of instability with resistance training. J. Strength Cond. Res..

[2] McBride J.M., Cormie P., Deane R. (2006). Isometric squat force output and muscle activity in stable and unstable conditions. J. Strength Cond. Res..

[3] McBride J.M., Larkin T.R., Dayne A.M., Haines T.L., Kirby T.J. (2010). Effect of absolute and relative loading on muscle activity during stable and unstable squatting. Int. J. Sports Physiol. Perform..

[4] Wahl M.J., Behm D. (2008). Not all instability training devices enhance muscle activation in highly resistance-trained individuals. J. Strength Cond. Res..

[5] Anderson K., Behm D. (2005). Trunk muscle activity increases with unstable squat movements. Can. J. Appl. Physiol..

[6] Andersen V., Fimland M.S., Brennset Ø., Haslestad L.R., Lundteigen M.S., Skalleberg K., Saeterbakken A.H. (2014). Muscle activation and strength in squat and bulgarian squat on stable and unstable surface. Int. J. Sports Med..

[7] Taylor J.B. (2011). Lower extremity perturbation training. Strength Cond. J..

[8] Behm D., Anderson K., Curnew R.S. (2002). Muscle force and activation under stable and unstable conditions. J. Strength Cond. Res..

[9] Hazell T.J., Kenno K.A., Jakobi J.M. (2010). Evaluation of muscle activity for loaded and unloaded dynamic squats during vertical whole-body vibration. J. Strength Cond. Res..

[10] Saeterbakken A.H., Fimland M.S. (2013). Muscle force output and electromyographic activity in squats with various unstable surfaces. J. Strength Cond. Res..

[11] Lawrence M.A., Carlson L.A. (2015). Effects of an unstable load on force and muscle activation during a parallel back squat. J. Strength Cond. Res..

[12] Willardson J.M., Fontana F.E., Bressel E. (2009). Effect of surface stability on core muscle activity for dynamic resistance exercises. Int. J. Sports Physiol. Perform..

[13] Aguilera-Castells J., Buscà B., Morales J., Solana-Tramunt M., Fort-Vanmeerhaeghe A., Rey-Abella F., Bantulà J., Peña J. (2019). Muscle activity of Bulgarian squat. Effects of additional vibration, suspension and unstable surface. PLoS ONE.

[14] Drinkwater E.J., Pritchett E.J., Behm D. (2007). Effect of instability and resistance on unintentional squat-lifting kinetics. Int. J. Sports Physiol. Perform..

[15] Vázquez-Guerrero J., Moras G., Baeza J., Rodríguez-Jiménez S. (2016). Force outputs during squats performed using a rotational inertia device under stable versus unstable conditions with different loads. PLoS ONE.

[16] Aguilera-Castells J., Buscà B., Arboix-Alió J., McEwan G., Calleja-González J., Peña J. (2020). Correlational data concerning body centre of mass acceleration, muscle activity, and forces exerted during a suspended lunge under different stability conditions in high-standard track and field athletes. Data Brief.

[17] Thiel D.V., Quandt J., Carter S.J.L., Moyle G. (2014). Accelerometer based performance assessment of basic routines in classical ballet. Procedia Eng..

[18] Johnston W., O’Reilly M., Coughlan G.F., Caulfield B. (2018). Inertial sensor technology can capture changes in dynamic balance control during the Y balance test. Digit. Biomark..

[19] Barbado D., Irles-Vidal B., Prat-Luri A., García-Vaquero M.P., Vera-Garcia F.J. (2018). Training intensity quantification of core stability exercises based on a smartphone accelerometer. PLoS ONE.

[20] Gopalai A.A., Senanayake S.M.N.A., Kiong L.C., Gouwanda D. (2011). Real-time stability measurement system for postural control. J. Bodyw. Mov. Ther..

[21] Kovacikova Z., Zemkova E., Neumannova K., Jelen M., Jelen K., Janura M. (2015). The role of lateral preference of lower limbs in a postural stabilization task. Neuroendocrinol. Lett..

[22] Goetschius J., Feger M.A., Hertel J., Hart J.M. (2018). Validating center-of-pressure balance measurements using the MatScan^®^ pressure mat. J. Sport Rehabil..

[23] Fort-Vanmeerhaeghe A., Bishop C., Buscà B., Aguilera-Castells J., Vicens-Bordas J., Gonzalo-Skok O. (2020). Inter-limb asymmetries are associated with decrements in physical performance in youth elite team sports athletes. PLoS ONE.

[24] Sánchez-Medina L., Pallarés J., Pérez C., Morán-Navarro R., González-Badillo J. (2017). Estimation of relative load from bar velocity in the full back squat exercise. Sport. Med. Int. Open.

[25] Robertson R., Goss F., Rutkowski J., Brooke L., Dixon C., Timmer J., Frazee K., Dube J., Andreacci J. (2003). Concurrent validation of the OMNI perceived exertion scale for resistance exercise. Med. Sci. Sport. Exerc..

[26] Cohen J. (1988). Statistical Power Analysis for the Behavioral Sciences.

[27] Hopkins W.G., Marshall S.W., Batterham A.M., Hanin J. (2009). Progressive statistics for studies in sports medicine and exercise science. Med. Sci. Sports Exerc..

[28] Koo T.K., Li M.Y. (2016). A guideline of selecting and reporting intraclass correlation coefficients for reliability research. J. Chiropr. Med..

[29] Moras G., Vázquez-Guerrero J. (2015). Force production during squats performed with a rotational resistance device under stable versus unstable conditions. J. Phys. Ther. Sci..

[30] Romero-Franco N., Martínez-López E.J., Lomas-Vega R., Hita-Contreras F., Osuna-Pérez M.C., Martínez-Amat A. (2013). Short-term effects of proprioceptive training with unstable platform on athletes’ stabilometry. J. Strength Cond. Res..

[31] Anderson K., Behm D. (2005). The impact of instability resistance training on balance and stability. Sport. Med..

[32] Krause D.A., Jacobs R.S., Pilger K.E., Sather B.R., Sibunka S.P., Hollman J.H. (2009). Electromyographic analysis of the gluteus medius in five weight-bearing exercises. J. Strength Cond. Res..

[33] Mausehund L., Skard A.E., Krosshaug T. (2019). Muscle activation in unilateral barbell exercises. J. Strength Cond. Res..

[34] Naclerio F., Larumbe-Zabala E. (2017). Relative load prediction by velocity and the OMNI-RES 0–10 scale in parallel squat. J. Strength Cond. Res..

[35] Panza P., Vianna J.M., Damasceno V.O., Aranda L.C., Bentes C.M., Novaes J., Behm D. (2014). Energy cost, number of maximum repetitions, and rating of perceived exertion in resistance exercise with stable and unstable platforms. J. Exerc. Physiol. Online.

[36] Brown A.F., Vianna J.M., Dias I.B., Miranda H.L., Neto G.R., Novaes J.S. (2014). Acute effects of different strength intensities on unstable and stable platforms on strength performance and subjective effort perception in bench press exercise. Med. Sport..

